# An Interesting Case of Tolosa-Hunt Syndrome in a Young Male

**DOI:** 10.1177/2324709616689478

**Published:** 2017-01-01

**Authors:** Ghulam Murtaza, Nicholas Konowitz, Hannah Lu, Anadil Faqah, Aneesh Kuruvilla

**Affiliations:** 1 UIC/Advocate Christ Medical Center, Oak Lawn, IL, USA; 2 Rosalind Franklin University of Medicine and Science, North Chicago, IL, USA

**Keywords:** headache, ptosis, Tolosa-Hunt syndrome, retro-orbital pain

## Abstract

Tolosa-Hunt syndrome is a rare disease with a limited number of cases reported in the literature. It typically presents with orbital pain associated with palsy of the third, fourth, or sixth cranial nerve. We present an interesting case of Tolosa-Hunt syndrome in a young male who responded well to high-dose steroids and in a few days had significant improvement in his retro-orbital pain and ocular movements.

## Introduction

Tolosa-Hunt syndrome (THS) is a rare steroid-responsive etiology for painful ophthalmoplegia. It was first described by Tolosa in 1954 and was explored further by Hunt when he published 6 cases of remittent unilateral retro-orbital pain accompanied by extraocular nerve palsies that responded well to steroid treatment. The estimated annual incidence is one case per million per year.^1^ It is characterized by unilateral orbital pain associated with paresis of one or more of the third, fourth, or sixth cranial nerve, commonly leading to diplopia. It is caused by idiopathic granulomatous inflammation.^2^ We present an interesting case of THS in a young male.

## Case Presentation

A 33-year-old Hispanic male with no relevant past medical history presented to our hospital with severe right-sided retro-orbital pain with right-sided ptosis and diplopia. The patient was at work 2 weeks prior when the onset of headache occurred. The headache began to worsen throughout the day. The patient went to a different hospital 3 days after onset of pain and was diagnosed with sinusitis and was sent home on antibiotic treatment. The pain continued to progress, and 1 week after onset of pain, the patient began to have ptosis and diplopia. He presented to another hospital where he was diagnosed with a cluster headache and was sent home with pain medications. The patient then visited an optometrist who referred him to an ophthalmologist due to the ptosis and diplopia. The ophthalmologist then urgently sent him to our hospital for magnetic resonance imaging (MRI) and follow-up.

In our emergency room, the patient endorsed deep right-sided retro-orbital pain that radiated down to his neck. He complained of neck stiffness but denied any symptoms of nausea or vomiting, recent upper respiratory symptoms, numbness, tingling, or weakness in any part of the body. He denied any sick contacts, recent travel, or work-related chemical exposures. The patient had no past medical or family history of headaches or migraines. On physical exam, the patient’s right eye was completely closed during initial consult. Adduction of the right eye past midline was limited to 25% of normal, and upward gaze was limited to 70% of normal. However, abduction, downward gaze, and vision were intact. Neurological exam was unremarkable. The patient was started on ketorolac 15 mg IV (intravenous) for pain control.

**Figure 1.**
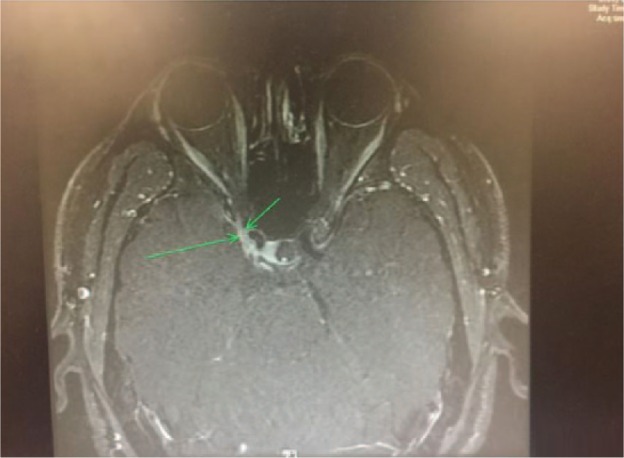
THS is characterized by mild focal asymmetric enhancement along the lateral wall of the right cavernous sinus just posterior to the orbital apex in the expected location of the right oculomotor nerve (green arrow).

**Figure 2.**
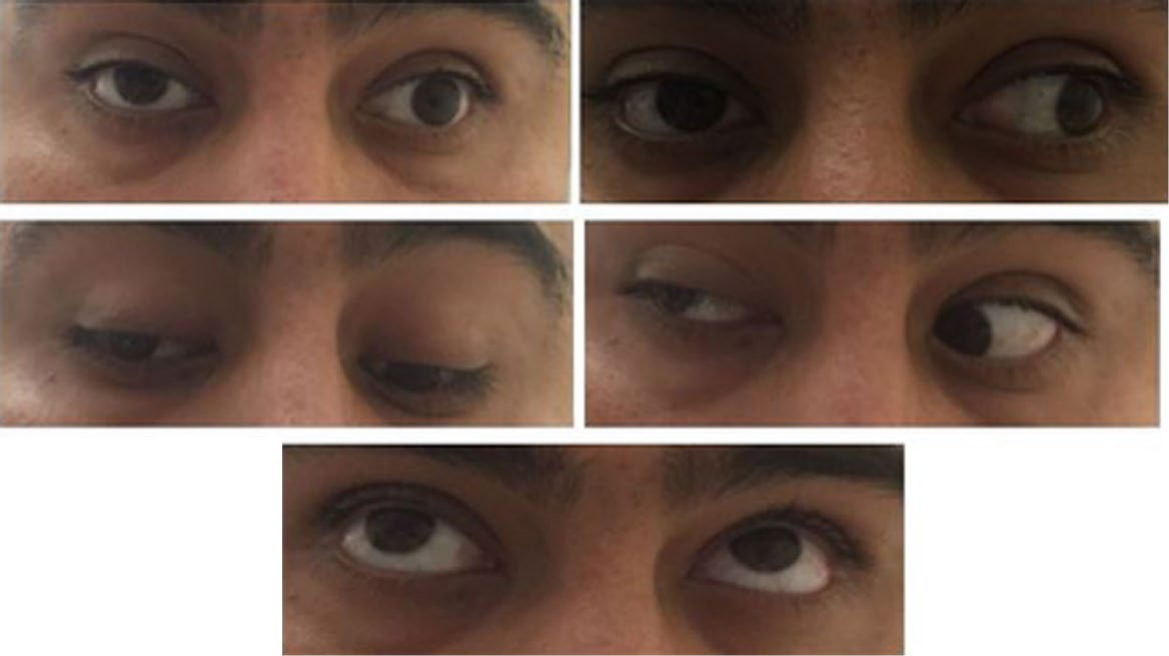
Neuro exam on day 1 of steroid treatment reveals right palpebral ptosis as well as paresis of the third cranial nerve characterized by defect in adduction (25% of normal) and upward gaze (70% of normal). Abduction and downward gaze were not affected in our patient.

MRI and magnetic resonance angiography (MRA) were done immediately, and it was determined that corticosteroid treatment would be held until an infectious etiology could be ruled out by lumbar puncture. MRA showed that the A1 segment was hypoplastic with no critical stenosis or aneurysm. MRI findings were suggestive of right oculomotor neuritis with a decrease in size of the right oculomotor nerve, increased surrounding fluid, and mildly increased contrast enhancement. An MRI of the orbit was recommended, which showed mild focal asymmetric enhancement along the lateral wall of the right cavernous sinus just posterior to the orbital apex in the expected location of the right oculomotor nerve (Figure 1). Calcium level was 8.8 mg/dL, ANA was negative, rheumatoid factor was negative, and erythrocyte sedimentation rate was not elevated. Chest X-ray did not show hilar adenopathy and was unremarkable otherwise. A complete infectious etiology was ruled out, which included the following: Lyme disease, herpes simplex virus, *Aspergillus, Coccidioides*, West Nile, syphilis, *Cryptococcus, Histoplasma/Blastomyces*. Complete vital panel was negative. A lumbar puncture was done, and cerebrospinal fluid (CSF) studies revealed normal glucose, normal protein, and one nucleated cell. CSF angiotensin-converting enzyme level was normal. However, a serum angiotensin-converting enzyme level was not checked. CSF cytology was negative; CSF IgG index was normal. Serum and urine protein electrophoresis were normal. The patient was immediately started on IV methylprednisolone 500 mg BID for 3 days. The patient’s pain and ocular movements began to marginally improve within 12 hours of the first dose (Figure 2).

**Figure 3.**
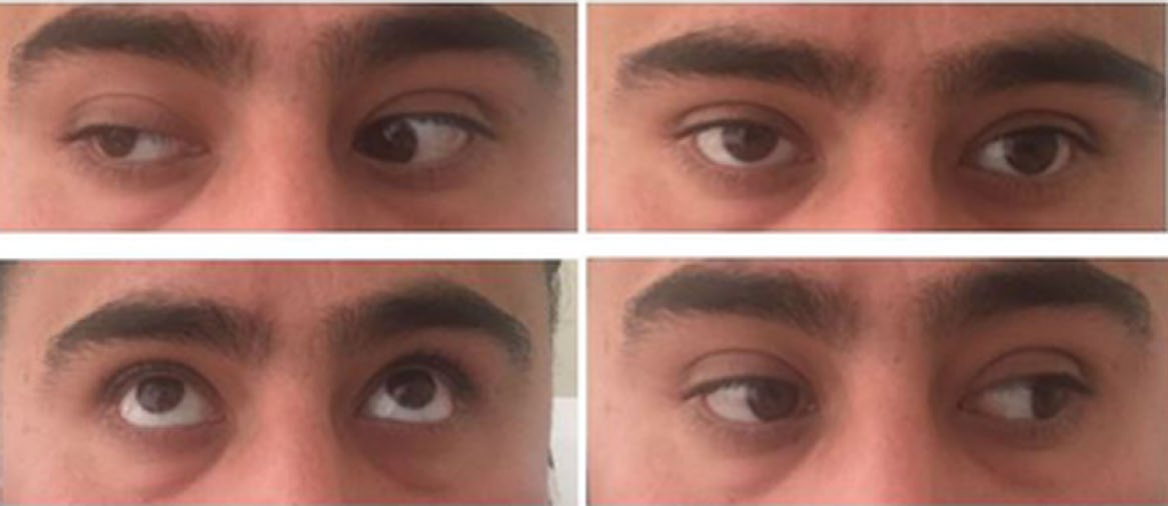
Neuro exam on day 3 of steroid treatment reveals improvement in right palpebral ptosis as well as improvement in paresis of the third cranial nerve characterized by improvement in adduction and upward gaze.

The patient continued IV steroids, and on day 3, he reported that his pain had completely resolved with significantly improved adduction of the right eye (Figure 3). The patient was released home on day 4 with almost complete normalization of his right eye adduction. The patient was discharged home on prednisone 60 mg daily for 7 days with follow-up with Neurology.

## Discussion

The International Headache Society guidelines for diagnosis of THS include the following: retro-orbital pain with an oculomotor palsy, granulomatous inflammation within the cavernous sinus, superior orbital fissure or orbit confirmed by MRI or tissue biopsy, the onset of the oculomotor palsy must be at the same time or within 2 weeks of the onset of orbital pain, and the pain must be localized around the ipsilateral brow and eye.^3^ THS follows a variable course that can last from days to weeks to months. Recurrences are common and can either be unilateral or bilateral.^4^ Other causes must be excluded by appropriate investigations. The retro-orbital pain has been shown to completely resolve within 72 hours of onset of steroid treatment, but the time needed for normalization of the cranial nerve palsies has been broad with an average of 26 days.^5^ Our patient met the aforementioned criteria and all infectious and autoimmune etiologies were ruled out making a diagnosis of THS more likely.

We believe this case demonstrates the importance of including THS as a differential diagnosis after all other diagnostic studies rule out more common etiologies for retro-orbital pain with oculomotor nerve palsy. Our patient visited 2 different emergency departments over a 2-week period and received separate diagnosis of sinusitis and cluster headaches, respectively, and only was sent to our emergency department after an ophthalmologist evaluation. Since THS responds well to steroid treatment, early detection is beneficial. Per the literature review, no standard dose for steroid treatment has been established. Our patient responded well to IV methylprednisolone 500 mg, and on day 3, he had almost complete resolutions of his pain and oculomotor nerve palsy. This response was faster than reported in the majority of cases.^5^ Studies have shown that younger patients are more likely to have a faster improvement in their oculomotor palsy, which could also explain our patient’s almost complete resolution of symptoms within 3 days of steroid treatment.^6^
